# Lecanemab in Practice: Real‐World Data from a 6‐Month Follow‐Up in Early Alzheimer’s Disease

**DOI:** 10.1002/alz70859_102792

**Published:** 2025-12-25

**Authors:** Noa Bregman, Talya Nathan, Dror Shir, Mori Hay‐Levy, Noa Trabulus, Aya Bar‐David, Galia Wolpe, Anan Abu‐Awad, Yifat Alcalay, Elissa Ash, Tamara Shiner

**Affiliations:** ^1^ Sagol School of Neuroscience, Tel Aviv University, Tel Aviv Israel; ^2^ Tel Aviv University, Tel Aviv Israel; ^3^ Neurological Institute, Tel Aviv Sourasky Medical Center, Tel Aviv Israel; ^4^ Tel Aviv Medical Center, Tel Aviv Israel; ^5^ School of Medicine, Tel Aviv University, Tel Aviv Israel

## Abstract

**Background:**

Alzheimer's disease (AD) is a progressive neurodegenerative disorder with a significant impact on patients and healthcare systems. Lecanemab, a monoclonal antibody targeting amyloid beta, has recently been approved for early‐stage AD, offering potential for disease modification. This report examines the demographic characteristics, treatment responses, and safety profile of early‐stage AD patients treated with Lecanemab at the Tel Aviv Medical Center (TLVMC), focusing on 6‐month follow‐up outcomes.

**Method:**

Since November 2023, Lecanemab has been administered to early‐stage AD patients at TLVMC. Data collected included baseline demographics, ApoE4 status, CSF biomarkers (t‐tau, p‐tau181, Aβ42), plasma p‐tau181 levels, and Mini‐Mental State Examination (MMSE) scores. Patients were monitored for amyloid‐related imaging abnormalities (ARIA), and cognitive outcomes were assessed at 6 months. This report summarizes data for patients who completed the 6‐month follow‐up by January 2025.

**Result:**

By January 2025, 45 out of 85 patients had completed the 6‐month visit. The mean age was 73 years (SD = 7.2), with 53% women and 51% ApoEε4 carriers. Mean baseline CSF biomarker levels (pg/ml): t‐tau = 580 (SD = 268), p‐tau181 = 194 (SD = 461), and Aβ42 = 333 (SD = 166). Plasma p‐tau181 levels (n=27) had a mean value of 32.3 pg/ml (SD = 10.5). The mean baseline MMSE score was 24.02 (SD = 2.59). Plasma p‐tau181 levels were significantly correlated with baseline MMSE scores (r = ‐0.429, p = 0.023). At 6 months, the mean MMSE score decreased to 22.48 (SD = 3.92), a significant decline of 1.47 points from baseline (*p* = 0.009). Younger patients (<75 years) showed a statistically significant decline in MMSE score (*p* = 0.007), while older patients (≥75 years) did not. Seven patients (15.5%) developed asymptomatic ARIA‐H during the first 6 months.

**Conclusion:**

This 6‐month follow‐up highlights Lecanemab's potential to preserve cognitive function in early‐stage AD, with a lower incidence of ARIA than reported in trials. These findings emphasize the importance of real‐world evidence in shaping clinical practice for disease‐modifying therapies.

